# Structure-based design and construction of a synthetic phage display nanobody library

**DOI:** 10.1186/s13104-022-06001-7

**Published:** 2022-03-29

**Authors:** Ernesto Moreno, Mario S. Valdés-Tresanco, Andrea Molina-Zapata, Oliberto Sánchez-Ramos

**Affiliations:** 1 Faculty of Basic Sciences, University of Medellin, Medellín, Colombia; 2 Grupo de Micología Médica y Experimental, Corporación para Investigaciones Biológicas (CIB), Medellín, Colombia; 3 Biological Sciences School, University of Concepción, Concepción, Chile

**Keywords:** Nanobody, Synthetic nanobody library, Phage display, Structure-based design

## Abstract

**Objective:**

To design and construct a new synthetic nanobody library using a structure-based approach that seeks to maintain high protein stability and increase the number of functional variants within the combinatorial space of mutations.

**Results:**

Synthetic nanobody (Nb) libraries are emerging as an attractive alternative to animal immunization for the selection of stable, high affinity Nbs. Two key features define a synthetic Nb library: framework selection and CDR design. We selected the universal VHH framework from the cAbBCII10 Nb. CDR1 and CDR2 were designed with the same fixed length as in cAbBCII10, while for CDR3 we chose a 14-long loop, which creates a convex binding site topology. Based on the analysis of the cAbBCII10 crystal structure, we carefully selected the positions to be randomized and tailored the codon usage at each position, keeping at particular places amino acids that guarantee stability, favoring properties like polarity at solvent-exposed positions and avoiding destabilizing amino acids. Gene synthesis and library construction were carried out by GenScript, using our own phagemid vector. The constructed library has an estimated size of 1.75 × 10^8^. NGS showed that the amino acid diversity and frequency at each randomized position are the expected from the codon usage.

**Supplementary Information:**

The online version contains supplementary material available at 10.1186/s13104-022-06001-7.

## Introduction

Nanobodies (Nbs) are single domain antibody fragments derived from heavy-chain antibodies found in members of the Camelidae family. They have several important advantages such as their small size, high solubility and stability. Remarkably, in spite of their monomeric binding region composed only of three hypervariable loops or CDRs (complementarity determining regions), Nbs can achieve affinities in the nanomolar and subnanomolar orders, just as clasical antibodies. Their modularity allows the easy generation of multivalent constructs for many different applications [1].

Antigen-specific Nbs are obtained mostly from inmune libraries, which requires animal immunization. In recent years, however, synthetic libraries are emerging as an attractive alternative for the selection of stable, high affinity Nbs, as recently reviewed by our group [2], while offering important advantages in terms of cost and speed. Two key features define a synthetic Nb library: framework selection and CDR design. A few recent works have relied on both sequence and structural data to define the CDR positions to be randomized, as well as the sets of amino acids (aa) to be introduced at those positions [3–9].

Here we describe the design and construction of a new synthetic nanobody library with a 14 amino acid-long CDR3, in which the three hypervariable loops were subjected to position-specific randomization schemes. The design follows a structure-based approach that seeks to maintain the high stability shown by the original framework-donor nanobody and increase the number of functional variants within the combinatorial space of mutations. To this aim we analyzed the structural role played by individual residues in defining CDR conformation or exposing their side chains for antigen binding.

## Main text

### Library design and construction

#### Framework selection

The synthetic library should be built on a scaffold shown to be highly stable, in particular to reducing conditions (as in the cytoplasm). Also, the framework should be capable of accepting many different CDRs while keeping high stability. Since current algorithms to predict protein stability still have important limitations [10], the safest option is to choose a camelid heavy-chain variable domain (VHH) framework already proven to possess the desired characteristics. We selected the “universal VHH framework” from the cAbBCII10 nanobody [11], which has been used previously as scaffold for the construction of Nb libraries [12–14] and not only its sequence is available, but also a few crystal structures containing CDR-grafted, the original and humanized versions of its framework (PDB entries 1ZMY [15], 3DWT and 3EAK [16], respectively.

#### CDR1 and CDR2 design

A main issue in our design was to keep the correct immunoglobulin domain folding and guarantee its stability, for which we thought important to keep the original cAbBCII10 internal packing as well as CDR1/CDR2 conformations. In consequence, both CDR1 and CDR2 were designed with a fixed length—the same as in cAbBCII10. Analysis of the available crystal structures allowed to assess the structural role played by different CDR residues and so guide the design. Buried CDR amino acids were kept as in the original nanobody since they are most likely important in maintaining the high framework stability. Therefore, only selected residues with surface-exposed side chains were subjected to randomization. Sequence variability was included by means of degenerate codons, represented in the sequence using the IUPAC degenerate base symbols [17]. Codon usage was tailored to limit the presence of hydrophobic amino acids at these positions. Cysteines were not allowed at any position. A few CDR aa found to be highly or relatively conserved in nanobody sequences, or thought to be important in holding CDR conformation in cAbBCII10, were also kept unchanged. It should be noted that our CDR definition follows that of Chothia and coworkers, which for CDR1 includes positions outside the classical Kabat CDR definition [18].

**Fig. 1:**
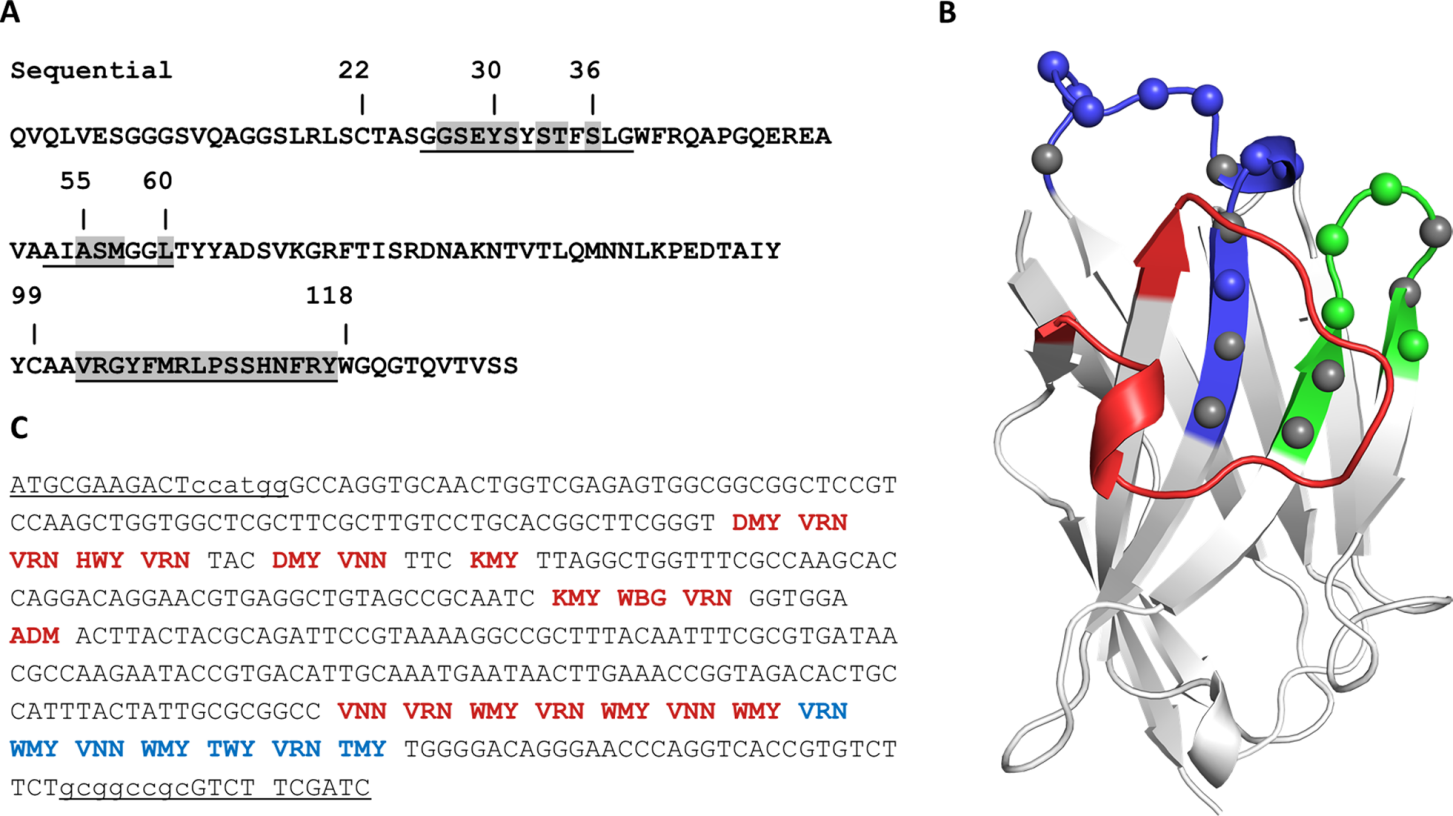
Sequence and structural basis for synthetic library design. **A** cAbBCII10 amino acid sequence showing the CDRs (underlined) and randomized positions (highlighted in gray). **B** cAbBCII10 structure (PDB: 3DWT). CDRs 1, 2 and 3 are colored in blue, green, and red, respectively. Colored spheres in CDRs 1 and 2 represent the randomized positions, while gray spheres represent CDR positions that were kept fixed. **C** The final nucleotide sequence for the whole nanobody, including the NcoI and NotI cloning sites

Figure 1A and B shows the sequence positions selected for randomization in each of the CDRs, marked on the sequence of the original cAbBCII10 and on its crystal structure. The rationale followed to define whether or not a CDR position is randomized, as well as the implemented codon variability for each position, is presented in Table 1. It is worth noting that, as for other libraries described in the literature, our design is based on premises involving a degree of subjectivity.

**Table 1:** Rationale for CDR design

Sequence position	Original aa	Degenerate codon	Coded aa	Analysis
CDR1				
26	Gly	–	Gly	Conserved (our own analysis). An outlier in the Ramachandran plot. Keep fixed
**27**	Gly	DMY	Ala:2 Asn:2 Asp:2 Ser:2 Thr:2 Tyr:2	An outlier in the Ramachandran plot. Randomize, but keep it small. The degenerate codon includes also Tyr
**28**	Ser	VRN	Arg:6 Asn:2 Asp:2 Gln:2 Glu:2 Gly:4 His:2 Lys:2 Ser:2	Surface-exposed side chain. Set it variable, but polar
**29**	Glu			
**30**	Tyr	HWY	Asn:2 His:2 Ile:2 Leu:2 Phe:2 Tyr:2	It is packed against the extremes of CDR1. Keep it mostly big and hydrophobic/aromatic
**31**	Ser	VRN	*see above*	Surface-exposed side chain. Set it variable, but polar
32	Tyr	–	Tyr	Buried aa playing an important packing role. Keep fixed
**33**	Ser	DMY	*see above*	It’s placed on an alpha turn, outside the binding site. Keep it mostly small, as the original aa
**34**	Thr	VNN	Ala:4 Arg:6 Asn:2 Asp:2 Gln:2 Glu:2 Gly:4 His:2 Ile:3 Leu:4 Lys:2 Met:1 Pro:4 Ser:2 Thr:4 Val:4	Points “upwards” at the base of CDR1. Set it very variable
35	Phe	–	Phe	Buried aa playing an important packing role. Keep fixed
**36**	Ser	KMY	Ala:2 Asp:2 Ser:2 Tyr:2	Support for CDR H3 (found also making a Cys bridge with CDR H3). Becomes buried by long CDRs 3. Small aa are commonly found, as well as Tyr
CDR2				
53	Ala	–	Ala	Within a β-sheet. Packed with the long CDR3. Keep fixed
54	Ile	–	Ile	Very conserved, buried aa playing an important packing role. Keep fixed
**55**	Ala	KMY	*see above*	Interacting with the bent CDR3 in the original Nb. Ser and Tyr are commonly found, as well as small aa
**56**	Ser	WBG	Arg:1 Leu:1 Met:1 Ser:1 Thr:1 Trp:1	Small aa side chains, like Ser and Thr, remain partially buried. The larger amino acids in the set would pack against CDR1 and the N-ter of CDR3
**57**	Met	VRN	*see above*	Surface-exposed side chain. Set it variable, but polar
58, 59	Gly	–	Gly	Conserved pair of residues, outliers in the Ramachandran plot. Keep fixed
**60**	Leu	ADM	Arg:1 Asn:1 Ile:2 Lys:1 Ser:1	Combines long charged aa. that can protrude upwards with small polar aa. Arg, Asn Ile and Ser are among the most frequently found aa at this position
CDR3				
VNN VRN WMY VRN WMY VNN WMY VRN WMY VNN WMY TWY VRN TMY				WMY: Asn:2 Ser:2 Thr:2 Tyr:2
				TWY: Phe:2 Tyr:2
				TMY: Ser:2 Tyr:2

IUPAC degenerate base symbols [14]:										
B = C, G, T﻿	H = A, C, T	M = A, C	R = A, G	V = A, C, G	Y = C, T	D = A, G, T	K = G, T	N = A, C, G, T	S = C, G	W = A, T

Numbers in bold correspond to CDR positions that were randomized

#### CDR3 design

For this library we chose a 14-long CDR3, which creates a “convex” binding site topology by bending over the flank that in classical antibodies is buried at the VH-VL interface [19, 20]. This represents an important difference as compared with a previously constructed library, carrying a 10 aa-long CDR3, as we analyze in the following section.

Three degenerate codons: VRN (coding for 8 polar or charged aa, and Gly), WMY (coding for Asn, Ser, Thr and Tyr in equal proportions) and the highly variable VNN (coding for 16 aa, excluding Cys, Phe, Trp and Tyr) alternate along most of CDR3, starting from its N-terminus (Table 1). The presence of five WMY codons places small aa (Asn, Ser, Thr) and Tyr every two positions, conferring conformational variability, while the high representation of Arg + Lys within the VRN and VNN codons increments their frequency in the alternating positions.

For the three C-terminal CDR3 residues (“n-2”, “n-1” and “n”) we chose particular degenerate codons to account for the aa frequency observed at these positions in nanobodies with long CDR3 loops, as well as for the structural role played by the particular amino acids found in the original cAbBCII10 Nb: Phe, Arg and Tyr (positions 115, 116 and 117, respectively).

For position “n” (117) we chose TMY, coding in equal proportions for Ser and Tyr—the two aa most commonly found at this position, with Tyr being the most frequent. In cAbBCII10, Tyr remains mostly packed with its OH group exposed. The side chain at the “n-1” position (116) is exposed to the solvent, so we chose the VRN codon. This residue holds also a backbone turn that bends down CDR3 on its C-terminal side. Finally, at the “n-2” position (115) in cAbBCII10, Phe is buried against a hydrophobic patch a the Nb flank, most likely playing an important structural role in the folding of CDR3. For this position we limited the repertoire to only two aa—Phe and Tyr (codon TWY). In fact, Tyr is the most frequent aa at this position. The final nucleotide sequence for the whole nanobody is shown in Fig. 1C.

#### Library construction and verification

Nanobody genes were cloned into our *ad-hoc* designed pMAC phagemid vector, whose map is shown in Additional file 1*.* This vector includes a pelB leader whose coding sequence contains a NcoI restriction site at the 3′ end. Other 3 unique restriction sites (EcoRI, BamHI and NotI) were added in tandem, followed by a sequence coding for a short linker (SGGGG) and a 6xHis tag, an amber stop codon and, finally, the M13 PIII protein. The synthesis of nanobody genes, library construction and next-generation sequencing (NGS) verification were carried out by GenScript (NJ, USA). Nb genes were cloned into the pMAC vector using the NcoI and NotI restriction sites, followed by transformation by electroporation of SS320 E. coli cells. The estimated library size was 1.75 × 10^8^.

**Fig. 2:**
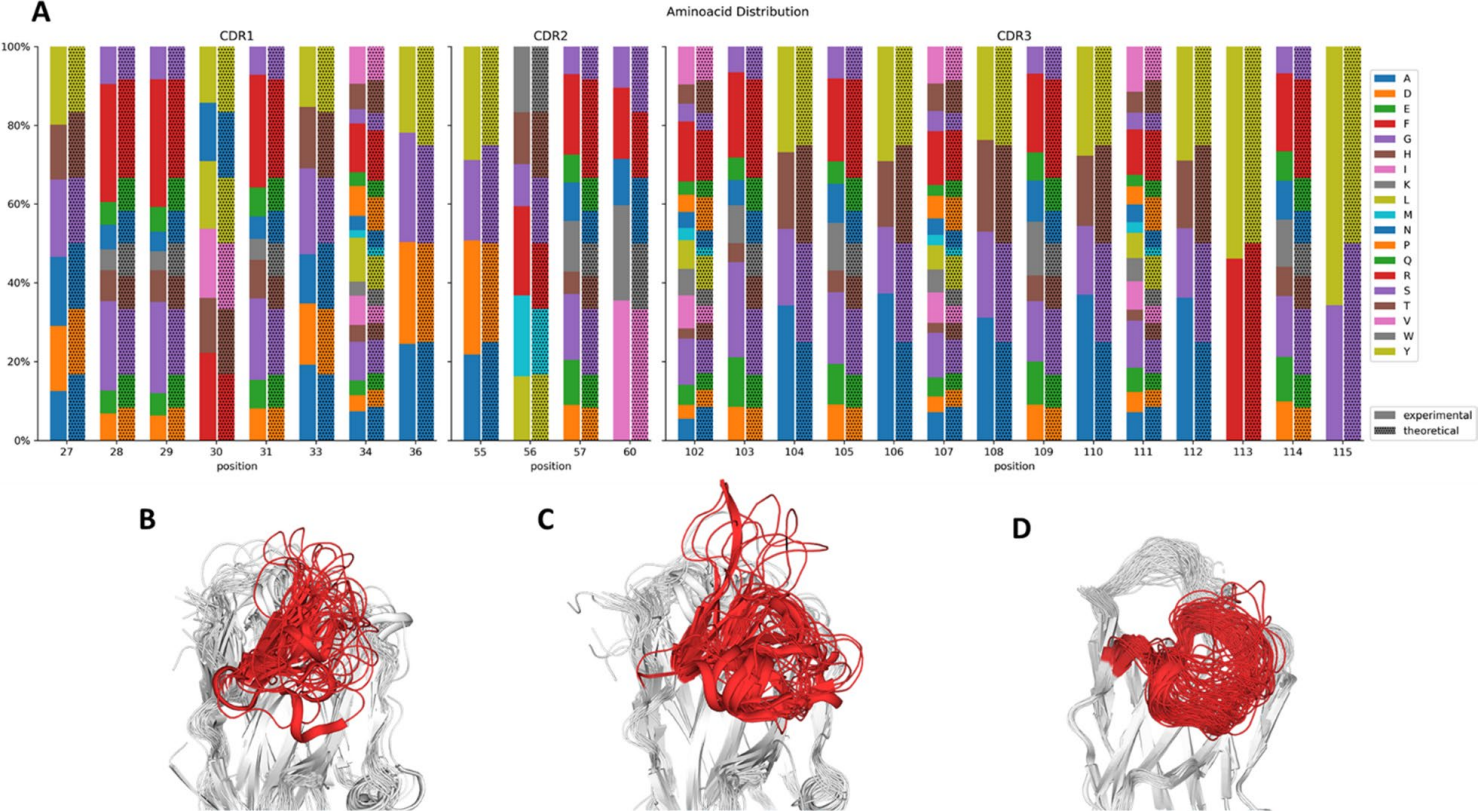
**A** Amino acid frequencies per randomized position for the constructed library. Each pair of bars represents the experimental frequencies obtained by NGS and the corresponding theoretical (designed) frequencies. **B**, **C** Structural superposition of 33 and 37 Nb crystal structures showing 10 aa-long and 14 aa-long CDR3 loops, respectively. **D** Structural superposition of 100 randomly selected library Nbs modeled with NanoNet

Figure 2A shows the obtained aa frequencies, as assessed by NGS, for all the randomized positions, together with the theoretically designed variability. At every position, the aa diversity is the expected from the codon usage, while the experimental aa frequencies are close to the theoretical ones.

#### Structural analysis of CDR3 conformations

In a previous work we constructed and tested against a panel of antigens a synthetic Nb library based also on the cAbBCII10 framework, but having an important structural difference with the one reported here: a shorter, 10 aa-long CDR3 (*manuscript in preparation*). It has been shown that different CDR3 lenghts create different binding site topologies [5]. Here we assessed these structural differences for the two implemented CDR3 lenghts (10 and 14 aa), using the nanobody structural data available in the Protein Data Bank (PDB) [21, 22], which has been rapidly growing in the last few years.

Currently, the PDB contains > 600 entries including a nanobody structure, either alone or in complex with an antigen [23]. Figure 2 shows the superposition of 33 and 37 nanobody crystal structures we found with either 10 (CDR3-10) or 14 aa-long (CDR3-14) CDR3 loops. CDR3-10 loops display a variety of conformations, going from the “upright” geometry adopted by most CDR H3 loops in classical antibodies to slightly or entirely bent loops (Fig. 2B). CDR-14 loops, on the other hand, adopt mostly bent conformations, with relatively few exceptions (Fig. 2C). Additionally, we used the NanoNet program [24] to construct structural models of 10,000 sequences of the CDR3-14 library, randomly generated based on the aa frequencies yielded by the degenerate codons used at each randomized position. Figure 2D shows the superposition of 100 randomly selected models, all of them displaying a bent CDR3 conformation. Overall, these analyses indicate that the two libraries display different binding site topologies, which may account for different “preferences” regarding antigen shape.

The huge amino acid variability introduced in the library creates also a huge variability in interaction networks between CDR amino acids, as well as between CDR and framework amino acids. For the longer CDR3-14 loops, the alternation of different hydrophobic and hydrophilic amino acids may define its particular bended conformation and also exert different effects in protein stability, because of the interactions of this loop with the framework flank that in conventional antibodies interact with the light chain VL domain.

#### Concluding remarks

By carefully selecting the positions to be randomized and tailoring the codon usage for each position, we sought to increase the number of functional combinations within the huge theoretical combinatory, keeping at particular positions amino acids that guarantee stability, favoring properties like polarity at solvent-exposed positions and avoiding destabilizing aa at certain positions. The design strategy presented here may be used on other stable frameworks with different CDR1-3 lengths.

## Limitations

We have yet to demonstrate that the constructed library can provide stable, high affinity nanobody binders for different antigens. This experimental work, though, has advanced for the CDR3-10 library, with very encouraging results (manuscript in preparation).

## Supplementary Information

**Additional file 1. **Map of the designed pMAC phagemid vector.

## Data Availability

The data generated or analyzed during this study were included in this article and its additional file.

## Abbreviations

Nb: Nanobody
aa: Amino acid(s)
CDR: Complementarity determining region
NGS: Next-generation sequencing
VHH: Camelid heavy-chain variable domain
