# Supplementary figures and images for: Structure-based design and construction of a synthetic phage display nanobody library
Source: BMC Res Notes. 2022 Mar 29;15:124. doi: 10.1186/s13104-022-06001-7 (PMC8966178; doi:10.1186/s13104-022-06001-7)

Map of the designed pMAC phagemid vector.

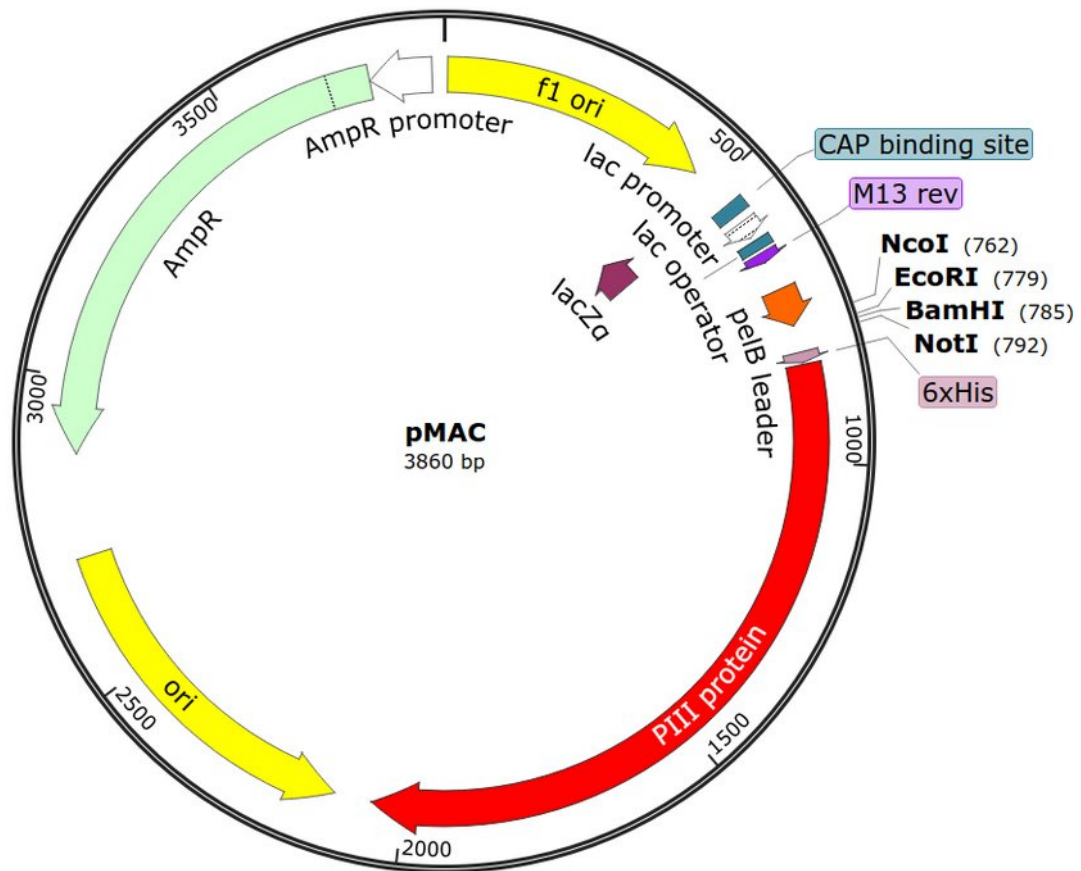

Supplement: Supplementary file 1 — Additional file 1. Map of the designed pMAC phagemid vector. [file 13104_2022_6001_MOESM1_ESM.pdf]
